# Signs of the 2009 Influenza Pandemic in the New York-Presbyterian Hospital Electronic Health Records

**DOI:** 10.1371/journal.pone.0012658

**Published:** 2010-09-09

**Authors:** Hossein Khiabanian, Antony B. Holmes, Brendan J. Kelly, Mrinalini Gururaj, George Hripcsak, Raul Rabadan

**Affiliations:** 1 Department of Biomedical Informatics, Columbia University College of Physicians and Surgeons, New York, New York, United States of America; 2 Center for Computational Biology and Bioinformatics, Columbia University College of Physicians and Surgeons, New York, New York, United States of America; 3 Department of Medicine, Columbia University College of Physicians and Surgeons, New York, New York, United States of America; Tulane University, United States of America

## Abstract

**Background:**

In June of 2009, the World Health Organization declared the first influenza pandemic of the 21^st^ century, and by July, New York City's New York-Presbyterian Hospital (NYPH) experienced a heavy burden of cases, attributable to a novel strain of the virus (H1N1pdm).

**Methods and Results:**

We present the signs in the NYPH electronic health records (EHR) that distinguished the 2009 pandemic from previous seasonal influenza outbreaks via various statistical analyses. These signs include (1) an increase in the number of patients diagnosed with influenza, (2) a preponderance of influenza diagnoses outside of the normal flu season, and (3) marked vaccine failure. The NYPH EHR also reveals distinct age distributions of patients affected by seasonal influenza and the pandemic strain, and via available longitudinal data, suggests that the two may be associated with distinct sets of comorbid conditions as well. In particular, we find significantly more pandemic flu patients with diagnoses associated with asthma and underlying lung disease. We further observe that the NYPH EHR is capable of tracking diseases at a resolution as high as particular zip codes in New York City.

**Conclusion:**

The NYPH EHR permits early detection of pandemic influenza and hypothesis generation via identification of those significantly associated illnesses. As data standards develop and databases expand, EHRs will contribute more and more to disease detection and the discovery of novel disease associations.

## Introduction

The first cases of infection by a novel swine-origin influenza A virus (H1N1pdm) [1] were reported in Mexico and the US in the spring of 2009 [2], [3]. By June 11, 2009, when the World Health Organization declared the first influenza pandemic of the 21^st^ century, 28,774 cases of laboratory-confirmed H1N1pdm infections, including 144 deaths, were reported in 74 countries [4], [5]. In New York City, by July 8, 2009, a total of 909 laboratory-confirmed cases had been hospitalized with H1N1pdm, of which 77% were under the age of 50 [6]. In addition, by the end of July, more than 27% of pediatric patients admitted to the city's New York–Presbyterian Hospital (NYPH) had a chief complaint of influenza-like illness (ILI) [7]. The spread of H1N1pdm continued into 2009–2010 influenza season and according to the Centers for Disease Control and Prevention (CDC), over 99% of the subtyped influenza cases in the season were found to be due to H1N1pdm.

NYPH, like other health care facilities around the US, increasingly uses electronic health records (EHRs) to document patient visits. The use of EHRs is set to rapidly increase over the next decade, driven by existing trends away from paper-based records and various government incentive programs [8], [9]. EHRs not only facilitate improvements in quality of care [10], [11], they also facilitate clinical research and epidemiological studies, particularly as they increase the availability of patients' longitudinal medical information [12], [13].

However, EHRs challenge researchers with the task of accurately identifying patients with a given medical condition [14], [15]. Detailed medical information about patients is found in textual discharge summaries authored by the physician responsible for their care that are only available for patients admitted to the hospital. Retrieving data requires employing natural language processing algorithms to turn the text into computable information [16]. Alternate sources of patient information include International Classification of Diseases diagnosis and procedure codes (ICD-10 and ICD-9), as well as the information from prescription orders and lab results. While the ICD codes are more easily extractable from EHRs, they are often entered by personnel not directly responsible for patients care, and so are not always accurate indicators of medical conditions [17], [18]. Datasets may also be discrepant due to dissimilar recording criteria and practices at different patient care sites, as a patient might have positive lab results for influenza, but not have the corresponding ICD code recorded at one site, and vice-versa at another site. Nevertheless, in cases of influenza, and at institutions like NYPH where influenza testing is routinely performed, ICD diagnoses can identify a minimal dataset, providing a lower bound for the actual number of flu patients [19], [20].

In this manuscript, we present the signs of the 2009 influenza pandemic evident in the EHR database collected at New York-Presbyterian Hospital in New York City from 2003 to 2009. These signs include an excess in the number of influenza patients, especially at expectedly low points of the flu season, and marked vaccine failure. In particular, the increase in the rate of influenza incidents is observed at a resolution as high as a zip code. We also investigate the differential age distribution of pandemic and seasonal influenzas, and analyze the EHRs for underlying health conditions that may be more prevalent among pandemic than seasonal influenza patients.

## Methods

The NYPH IRB protocol for this project was marked as Non Human Subject Research and thus was exempt from the requirement of formal approval by the IRB. The NYPH EHR was de-identified in accordance with the HIPPA regulations and all data that could identify patients was removed before the study was commenced. This limited dataset includes various tables containing the demographics information, diagnoses and procedures data (indicated by their respective ICD-9 codes), lab results, and lists of prescription orders.

Considering the previously discussed inaccuracies of ICD coding, we selected our set of patients based on the general ICD-9 code for influenza (487) and its subcategories. At NYPH influenza testing is routinely performed and in particular, it was mandated for all patients admitted to the hospital with ILI during the 2008-2009 season. The number of patients selected, therefore, represents the lower bound for the actual number of influenza patients who visited NYPH.

We assume that patients diagnosed with influenza after May of 2009 were symptomatically ill with H1N1pdm, identifying them as pandemic influenza patients. Similarly, patients diagnosed with influenza before May 2009 are identified as seasonal influenza patients.

To identify patients vaccinated with influenza vaccine, we refer to the ICD-9 procedure code 99.52 (prophylactic vaccination against influenza) and 5 NYPH internal Medical Entity codes from procedure tables. Using these codes, we are able to identify the patients who received the influenza vaccine in 2003–2009 seasons. At NYPH, vaccines are administered as per New York City Department of Health guidelines: the seasonal influenza vaccine is recommended for pregnant women, health care workers, anyone 6 months through 18 years of age, anyone 50 years or older, anyone caring for infants less than 6 months of age, and anyone with an underlying health condition that increases the risk of complications from influenza (asthma, heart disease, diabetes, etc.) [21]. Of note, the codes used to identify vaccination events capture only vaccination performed at NYPH – not vaccination reported by patients as having occurred elsewhere. We also exclude any vaccinations against H1N1pdm, as their analysis belongs to the 2009–2010 influenza season. We finally define an incident of vaccine failure when a vaccinated patient is diagnosed with influenza during the same season, at least 30 days after the inoculation.

To find the excess in number of patients in each age group per season, relative to the total number of influenza patients, we define Age Dependent Risk (ADR) by
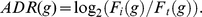



Here, *F_i_*(*g*) is the normalized number of influenza patients of age group *g* in season *i*, relative to the total number of patients in the season, and *F_t_*(*g*) is the normalized number of seasonal influenza patients of age group *g* in all seasons, relative to the total number of patients.

For every influenza patient, we collected the ICD-9 diagnoses codes recorded in various periods of some months before and after the influenza diagnosis. For each time interval, we computed the one-tail hypergeometric probability distribution to find whether there are any statistically significant differences in the prevalence of medical conditions in pandemic versus seasonal patients. Next, we calculated the False Discovery Rate (FDR) to adjust the p-values given the multiple hypotheses tested. FDR for probability *p_0_*, is defined as
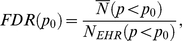



where *N_EHR_* is the number of hypotheses with p-values smaller than *p_0_* derived from the EHR pandemic and seasonal datasets. 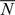 is the expected number of such hypotheses, calculated via a bootstrapping method; fixing the number of patients in each dataset, we randomly assigned patients to the bootstrapped pandemic or seasonal datasets, without changing their sets of diagnoses. The one-tail hypergeometric probabilities for each diagnosis were then recalculated and the two bootstrapped datasets were compared. We repeated the bootstrapping step 2000 times (approximately the number of patients in each dataset), and found 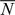 as the average number of p-values less than *p_0_* per bootstrapped dataset.

## Results

Employing the specific ICD-9 code for influenza (487 and its subcategories) to select the influenza patients of the past 6 seasons between 2003 and 2009 (from September 2003 to September 2009), we identified 3368 distinct patients for whom the majority of the diagnoses are recorded as “Influenza with other respiratory manifestations” (ICD-9 code 487.1). No influenza strain subtype is available in this dataset.

**Figure 1:**
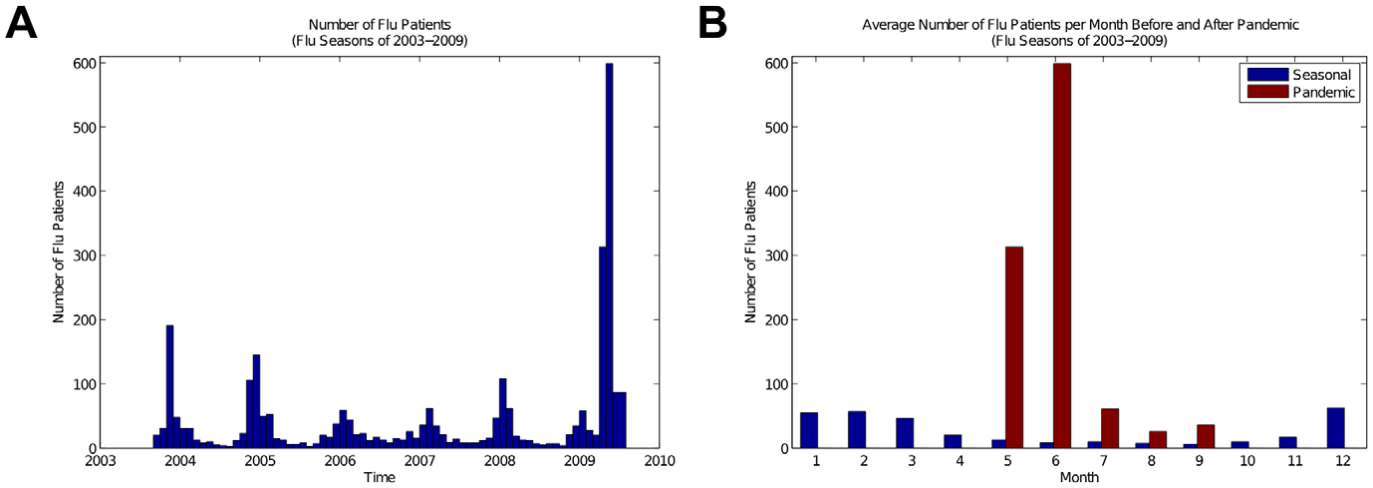
An increase in the number of influenza patients. (A) Number of patients between 2003–2009 influenza seasons. A marked increase in the number of recorded influenza cases is apparent by the beginning of May 2009. (B) Average Number of patients per month, before and after the H1N1pdm pandemic. Between 2003 and 2009, seasonal influenza cases consistently peaked from December to March, whereas the peak of the pandemic occurred in May and June 2009.

Figure 1A shows the number of flu patients during this period, with a substantial increase in the number of patients after May 2009, when the H1N1pdm epidemic started in New York City. We also observe that the increase in flu patients during the months of the pandemic occurred when the average number of seasonal flu patients per month typically falls (Fig. 1B).

**Figure 2:**
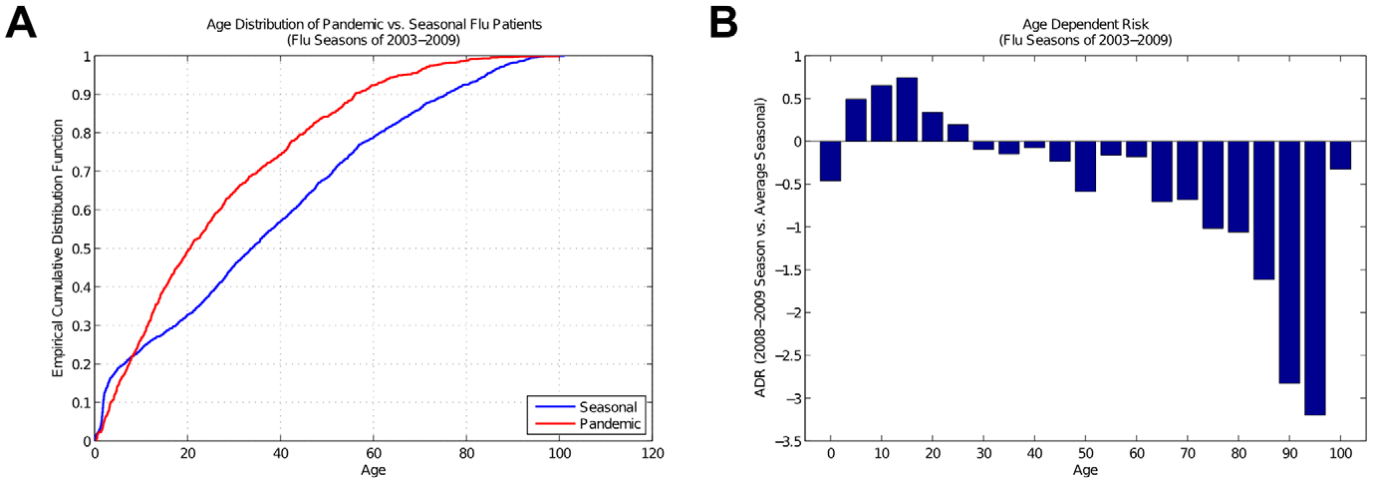
Age Distribution of Pandemic vs. Seasonal influenza Patients. (A) The differential age distribution of the pandemic influenza cases compared to seasonal cases is statistically significant, according to Mann-Whitney (*p*<0.001) and Kolmogorov-Smirnov (*p*<0.001) nonparametric tests. (B) Age Dependent Risk for influenza patients of 2008–2009 season versus the whole dataset. An increase in the number of patients between ages of 5 and 25 and a distinct decrease in the number of patients older than 60 is observed.

We compared the seasonal and pandemic influenza patients regarding their age and found substantial dissimilarities in the mean ages (36 years in seasonal vs. 26 years in pandemic patients) and median ages (33 years in seasonal vs. 20 years in pandemic patients). Figure 2A shows the respective age distributions' Empirical Cumulative Distribution Functions, for which both nonparametric Mann-Whitney (*p*<0.001) and Kolmogorov-Smirnov (*p*<0.001) tests indicate statistically significant difference. These tests respectively compare the two cumulative distributions via their ranking difference and their maximum difference. Of note, we did not find a statistically significant difference between the gender distributions of the seasonal and the pandemic influenza patients.

We also calculated Age Dependent Risk (ADR) for influenza patients of 2008–2009 season, in which H1N1pdm was the predominant strain, versus the whole dataset, as a measure of the expected age distribution. We found an increase in the number of patients between ages of 5 and 25 and a distinct decrease in the number of patients older than 60 (Fig. 2B).

**Figure 3:**
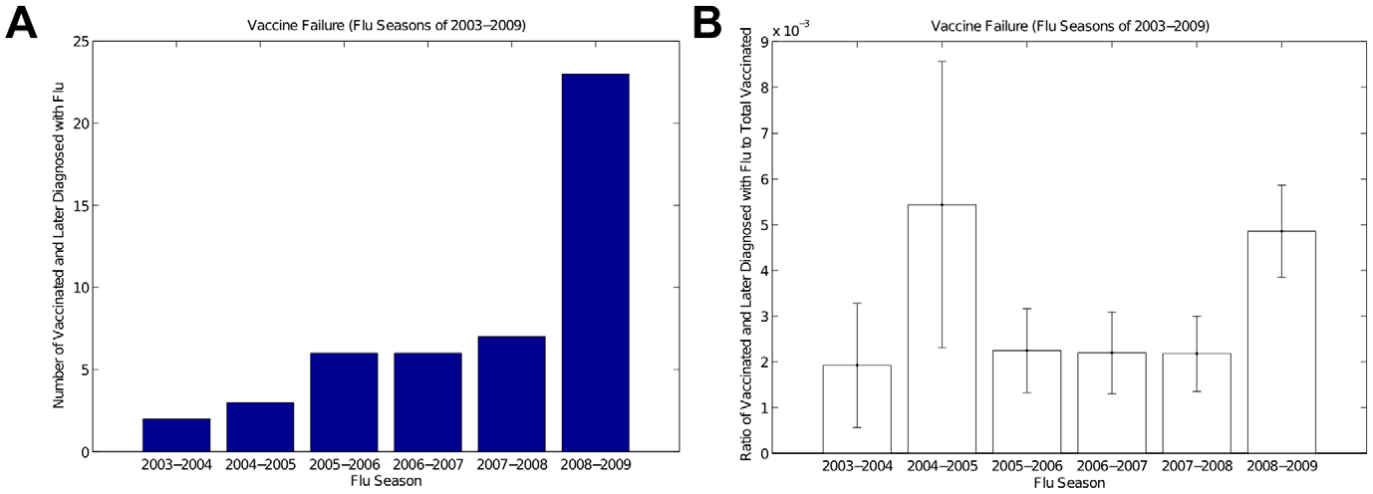
Vaccine Failure in 2008–2009 influenza season. (A) The increase in the number of patients who received the vaccine and later were diagnosed with influenza and (B) their ratio to the total number of recorded vaccinations are statistically significant in 2008–2009 season compared to the previous seasons. (There were low numbers of EHR-recorded cases of vaccination during the 2004–2005 season, which is consequently seen in the large error-bars.)

Furthermore, we collected a set of patients who are recorded as being vaccinated between the 2003–2004 and 2008–2009 influenza seasons. This set is not complete, as vaccinations were not routinely documented in the NYPH EHR during these time intervals; however, we were able to identify patients with influenza diagnoses given in the season when the vaccination occurred – patients who point to incidents of vaccine failure. Figure 3 shows the ratio of these influenza patients relative to the total number of vaccinated individuals in each season. The ratio of patients who received the vaccine and later were diagnosed with influenza in the 2008–2009 season is substantially increased compared to the previous seasons. (It should be noted that there were low numbers of EHR-recorded cases of vaccination during the 2004–2005 season, which is consequently seen in the large error-bars in Figure 3B.)

Moreover, we employed the longitudinal diagnosis data available for the influenza patients in our dataset to identify medical conditions associated with pandemic influenza (according to their ICD-9 coding). Prior analyses have utilized ICD-9 diagnoses given at the same time as the diagnosis of interest to construct mortality risk models [22], [23]; we present the set of associated diagnoses found in the database during a variable time window (Table 1 and Table S1). Increasing the size of the interval increases the number of diagnoses associated with seasonal and pandemic influenza patients, and so increases the number of hypotheses tested. P-values listed in Table 1 are the one-tail hypergeometric probabilities, along with their False Discovery Rate (FDR) derived via a bootstrapping method, correcting for the multiple hypotheses tested.

**Table 1:** ICD-9 codes associated with pandemic influenza, compared to seasonal.

Inquiry Interval (time pre/post flu)	ICD9 Codes *	Diagnoses	P-values †	FDR ‡	Ratio (Pandemic/Seasonal)
8 months (6/2)	**493.9**	**Asthma, unspecified**	**<0.001**	**<0.001**	**2.06**
	**V12.61**	**Personal history of pneumonia**	**<0.001**	**0.013**	**6.31**
	787.2	Dysphagia, unspecified	0.001	0.055	5.41
	348.39	Other encephalopathy	0.002	0.064	8.11
	110.9	Dermatophytosis	0.002	0.097	6.01
	315.8	Other specified delays in development	0.004	0.116	3.60
	709.8	Other specified disorders of skin	0.004	0.128	7.21
	345.9	Epilepsy unspecified	0.005	0.121	2.58
	789.59	Other ascites	0.006	0.173	10.8
	V22.2	Pregnant state incidental	0.009	0.212	2.03
	262	Other protein-calorie malnutrition	0.010	0.219	6.31
	338.29	Other chronic pain	0.010	0.219	6.31
	599.7	Hematuria, unspecified	0.012	0.251	4.81
	315.9	Other specified delays in development	0.013	0.243	2.92
	577	Acute pancreatitis	0.014	0.265	3.60
6 months (5/1)	**493.9**	**Asthma, unspecified**	**<0.001**	**<0.001**	**2.05**
	**787.2**	**Dysphagia, unspecified**	**<0.001**	**0.002**	**19.8**
	**V12.61**	**Personal history of pneumonia**	**<0.001**	**0.021**	**5.86**
	**110.9**	**Dermatophytosis**	**0.001**	**0.022**	**9.01**
	348.39	Other encephalopathy	0.002	0.054	8.11
	315.8	Other specified delays in development	0.004	0.106	3.60
	709.8	Other specified disorders of skin	0.004	0.117	7.21
	789.59	Other ascites	0.006	0.151	10.8
	262	Other protein-calorie malnutrition	0.010	0.207	6.31
	379.92	Swelling or mass of eye	0.010	0.207	6.31
	V22.2	Pregnant state incidental	0.011	0.208	2.23

*ICD codes and diagnoses lists include all p-values <0.015, excluding symptoms of influenza infection, and procedure-related supplemental (V) or external injury (E) codes (see Supplemental Material Table S1 for additional time windows and Table S2 for all excluded ICD codes and diagnoses)

† one-tail hypergeometric p-values, uncorrected;

‡ false discovery rate (FDR) described in Methods — significant at FDR <0.05 (bolded)

Though the associations listed in Table 1 must be interpreted with caution, we found associations for which the null hypothesis is not rejected (i.e., FDR<0.05) and their significance hold through multiple intervals of inquiry. In particular, we found significantly more pandemic flu patients with diagnoses associated with asthma and underlying lung disease. However, pregnancy and obesity, preliminarily reported as potential risk factors [24], [25], do not have statistically significant associations with pandemic influenza in the NYPH EHR. (Also, see Table S2 for all excluded ICD codes and diagnoses.)

**Figure 4:**
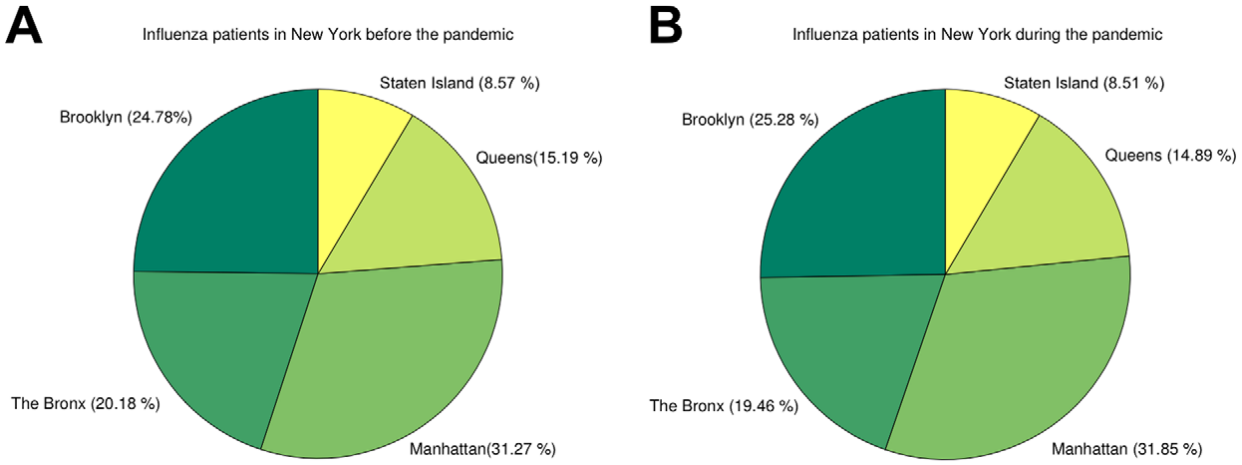
NYPH serves all five boroughs of New York City, mostly Manhattan and also some parts of the Bronx. Although cases of patients with influenza from Staten Island, Brooklyn, and Queens are present, they appear to be fairly isolated cases and do not reflect the movement patterns of the New York populace, as seen (A) before and (B) during the pandemic.

**Figure 5:**
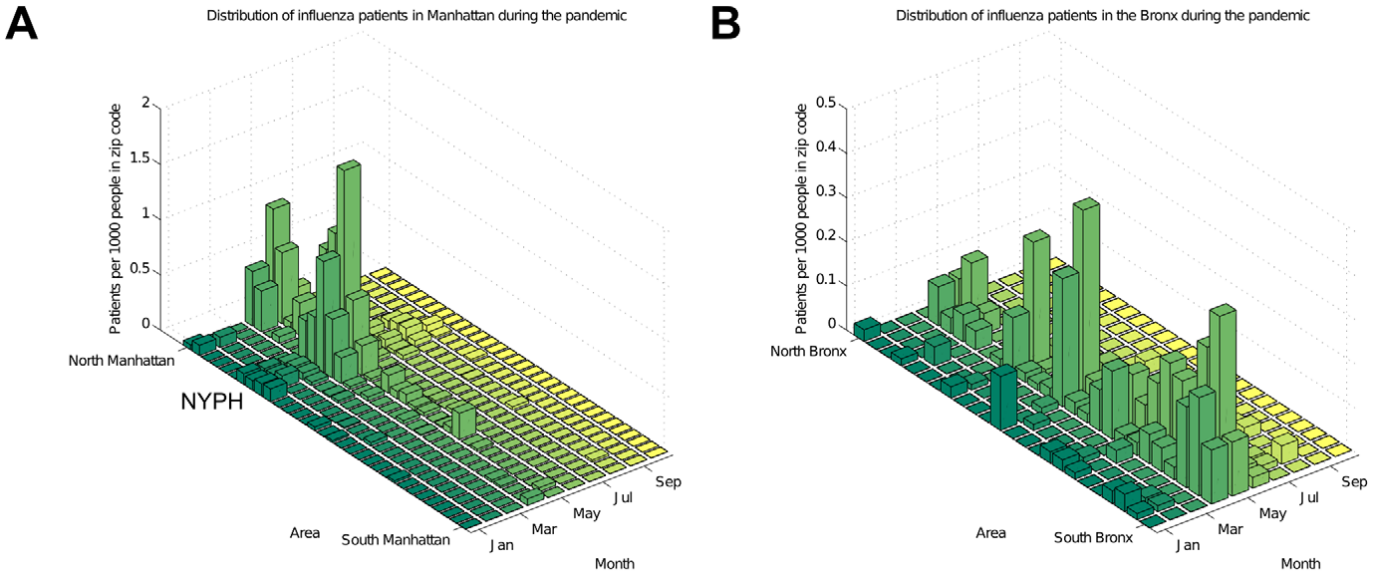
Distribution of influenza patients in Manhattan and the Bronx during 2009. (C) Distribution of influenza patients during 2009, mostly visiting from northern Manhattan. (D) Interestingly, we find that the number of influenza patients from south parts of the Bronx also increased rapidly during the spring of 2009, peaking in April and May corresponding to the incidence pattern of H1N1pdm. Patient rates are calculated per 1000 people in each zip code, according to the 2000 census population numbers. From left to right, the zip codes are ordered from north to south in Manhattan and the Bronx.

NYPH serves all five boroughs of New York City, although it is predominantly visited by people from Manhattan. EHRs provide demographic information allowing patient groups in specific areas to be studied. This type of information is especially useful during an epidemic or a pandemic, since it allows the source of the outbreaks to be discerned [26]. Available data however, typically monitors populations over large geographic areas such as country, state, or city (for example, CDC Flu homepage [27], WHO FluNet [28], [29], and European Influenza Surveillance Network [30]). Figures 4A and 4B show the distribution of influenza patients before and during the 2009 pandemic. The distribution of patients remains very similar during both periods. This shows that the number of people with symptomatic influenza rose across all five boroughs at similar rates, indicating that the whole city was affected. Although NYPH is mostly visited by people from the northern Manhattan neighborhood surrounding it, we find that the number of influenza patients from the Bronx increased rapidly during the spring of 2009, peaking in April and May, corresponding to the incidence pattern of H1N1pdm. The NYPH EHR is therefore capable of tracking diseases at a resolution as high as particular zip codes (Figs. 5A and 5B).

## Discussion

NYPH began using an EHR database in 1988 and has progressively increased the use of such systems ever since so that the primary method of data entry in the hospital is now electronic. The amount of data entered into the database per year has been increasing at an exponential rate since 1990, doubling every 8 years; by the end of 2008, more than 700 million data entries (notes, reports, batteries of test, etc.) had been entered into the system. The number of entries per person has also been increasing linearly, with an average of 300 entries generated per patient in 2008.

EHRs represent a new set of tools to assist the early identification of pandemic illness, and the NYPH records already shows several signs distinguishing H1N1pdm from prior seasonal influenza outbreaks. A marked increase in the number of recorded influenza cases is apparent by the beginning of May 2009 (Fig. 1A). The trend in average number of influenza diagnoses per month also distinguishes H1N1pdm from seasonal influenza (Fig.1B): according to the NYPH EHR, during the past 6 years, seasonal influenza cases consistently peaked from December to March, whereas the peak of the pandemic occurred in May and June 2009.

EHRs not only help to identify a novel disease outbreak, but they do so with high geographic resolution [26]. The distribution of patients in the five boroughs of New York City before and during the pandemic (Figs. 4A and 4B) suggests that patterns of EHR usage remain fairly consistent. If more people get influenza, more patients will come to NYPH, so that the records reflect the trends in a large part of New York City. In particular, we observe a substantial increase in the number of visits by influenza patients from Manhattan and the Bronx who were diagnosed during the pandemic months. Figure 5 shows the rate of influenza patients in Manhattan and the Bronx in each zip code per 1000 people according to the 2000 census population numbers, further demonstrating that the EHRs' geographic information is valuable for tracking the spread of the disease and as a potential predictor of future outbreaks.

Moreover, the NYPH EHR confirms preliminary reports indicating that a significant majority of pandemic influenza patients were younger than 60 years old [31]. Figure 2A shows the Empirical Cumulative Distribution Functions of the age distributions of the seasonal influenza patients of the past 6 seasons and the influenza patients of the 2009 pandemic. We observe that the differential age distribution of pandemic influenza cases compared to seasonal cases is statistically significant (*p*<0.001). Furthermore, Figure 2B shows the Age Dependent Risk (ADR) of pandemic influenza versus seasonal, where there is a substantial increase in the number of pandemic influenza patients aged between 5 and 25 and a marked decrease in the number of pandemic influenza patients aged older than 65. These results are in accordance with the preliminary results in New York City [6], [25] and nation-wide [32]. However, this feature of the EHR data may not suffice as a means to distinguish H1N1pdm from seasonal influenza because a similar differential age distribution has also been observed between seasonal strains, where the symptomatic influenza due to seasonal H1N1 is distributed mainly in a younger population relative to seasonal H3N2 [33], [34].

We also identify signs of vaccine failure in NYPH EHR (Fig. 3), which further help to distinguish H1N1pdm from prior seasonal influenza outbreaks. Figure 3 shows the substantial increase in the number of vaccine failures in 2008–2009 season. However, vaccine failure, like age distribution, may be insufficient alone to identify pandemics; seasonal influenza vaccines are not always designed effectively, especially when an infecting influenza virus is antigenically dissimilar to the expected strains that are included in the vaccine design [35]. This was the case in the 2003–2004 season (Fig. 1A), when the vaccine failed for adults [36], although it was partially effective for those younger than 9 years of age [37].

The limited recording of vaccinations in the NYPH EHR raises the issue of data quality – there is no doubt that whilst NYPH continues the transition from paper to electronic health records, its database will remain incomplete. The cases of pandemic and seasonal influenza analyzed here must be regarded as a minimal data set, perhaps only partially representative of the larger set of influenza cases actually treated at NYPH. Nevertheless, statistically significant associations between pandemic influenza and various comorbid conditions can be detected in the NYPH EHR. In Table 1, we propose a method for variable-interval inquiries of EHRs. Longer intervals are less specific, but are necessary to ascertain associations with time-sensitive diagnoses (such as pregnancy, which was identified by the CDC early in the course of the pandemic as a potential risk factor for H1N1pdm), whereas shorter intervals of inquiry yield fewer ICD-9 codes [25].

**Figure 6:**
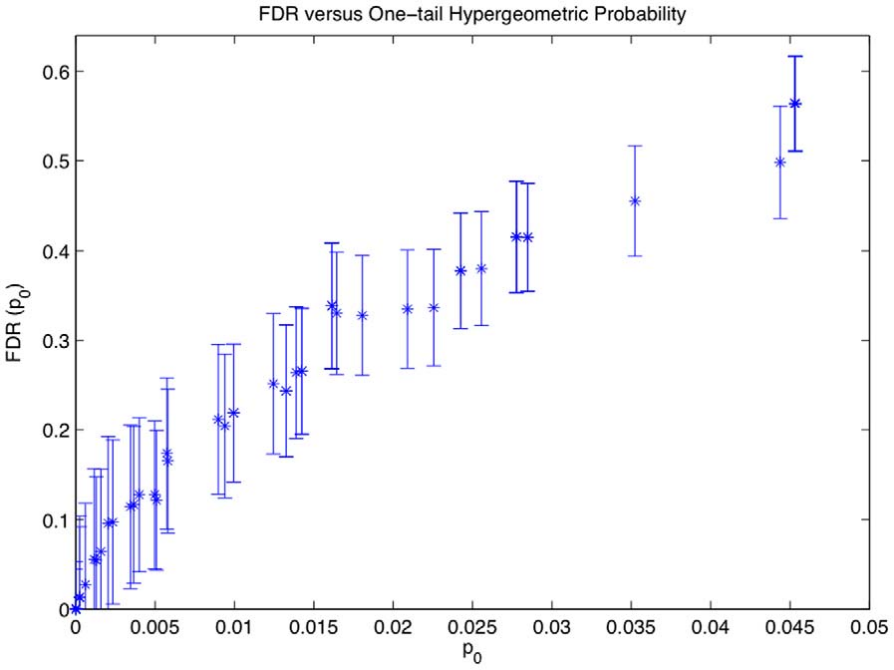
The False Discovery Rate (FDR) versus the one-tail hypergeometric probabilities, *p_0_*, for p-values less than 0.05. The error-bars are the standard errors for the time interval of 6 months before and 2 months after the influenza diagnosis. FDR is the ratio of the expected number hypotheses with p-values less than *p_0_*, derived from bootstrap datasets, to the number of such hypothesis in the EHR pandemic and seasonal datasets.

When the pandemic versus seasonal comparison probabilities for the diagnoses in each time interval are calculated, we find more than 70% of the hypotheses with p-values larger than 0.5. These high p-values are due to diagnoses with low number of recorded patients, which could never reach a high level of statistical significance. Given the high number of associations with high p-values, traditional corrections for statistical significance in situations of multiple hypotheses testing (such as the Bonferroni or Benjamini-Hochberg methods) are not applicable – they falsely increase the number of tested hypotheses, reducing the number of significant candidates. Therefore, to correct for multiple hypothesis testing while maintaining the structure of the dataset, we calculate the False Discovery Rates (FDRs) via a bootstrapping method (Fig. 6).

The associations for which the null hypothesis is not rejected (i.e., FDR<0.05) that persist through multiple intervals of inquiry (e.g., asthma, prior pneumonia, dysphagia) might be of clinical interest and help to confirm or refute preliminary reports of H1N1pdm (Table 1). Of note, pregnancy and obesity (potential risk factors identified in such reports [24], [25]) do not have statistically significant associations with pandemic influenza in the NYPH EHR. Notably, this analysis excludes considerations of disease severity.

EHRs allow unprecedented access to large sets of patients' longitudinal medical information and allow analysis of such information to occur in near real-time. In particular, substantial excess in the number of patients, especially at a period outside of the normal influenza season, and significant vaccine failure, readily evident in EHRs, are clear indicators of a circulating strain to which the public lacks immunity. Even the sparse data available at NYPH permits early detection of pandemic influenza and hypothesis generation via identification of those significantly associated illnesses, demonstrating the benefits that EHRs might extend to population health. As data standards develop and databases expand, EHRs will contribute more and more to disease detection and the discovery of novel disease associations.

## Supporting Information

Table S1 ICD-9 codes associated with pandemic influenza, compared to seasonal (additional time windows).
(0.09 MB DOC)

Table S2 Excluded ICD-9 codes associated with pandemic influenza, compared to seasonal.
(0.13 MB DOC)

## References

[1] Trifonov V, Khiabanian H, Rabadan R (2009). Geographic dependence, surveillance, and origins of the 2009 influenza A (H1N1) virus.. N Engl J Med.

[2] Dawood FS, Jain S, Finelli L, Shaw MW, Lindstrom S (2009). Emergence of a novel swine-origin influenza A (H1N1) virus in humans.. N Engl J Med.

[3] Centers for Disease Control and Prevention (2009). Swine influenza A (H1N1) infection in two children--Southern California, March-April 2009.. MMWR Morb Mortal Wkly Rep.

[4] World Health Organization (2009). World now at the start of 2009 influenza pandemic.. http://www.who.int/mediacentre/news/statements/2009/h1n1_pandemic_phase6_20090611/en/index.html.

[5] World Health Organization (2009). Influenza A(H1N1) - update 47.. http://www.who.int/csr/don/2009_06_11/en/index.html.

[6] New York City Department of Health and Mental Hygiene (2009). New York City Department of Health and Mental Hygiene health alert No. 27: pandemic (H1N1) 2009 influenza update, revised reporting requirements and testing procedures.. http://www.nyc.gov/html/doh/downloads/pdf/cd/2009/09md27.pdf.

[7] Miroballi Y, Baird JS, Zackai S, Cannon JM, Messina M (2010). Novel influenza A(H1N1) in a pediatric health care facility in New York City during the first wave of the 2009 pandemic.. Arch Pediatr Adolesc Med.

[8] Steinbrook R (2009). Health care and the American Recovery and Reinvestment Act.. N Engl J Med.

[9] Hendy J, Reeves BC, Fulop N, Hutchings A, Masseria C (2005). Challenges to implementing the national programme for information technology (NPfIT): a qualitative study.. BMJ.

[10] DesRoches CM, Campbell EG, Rao SR, Donelan K, Ferris TG (2008). Electronic health records in ambulatory care--a national survey of physicians.. N Engl J Med.

[11] Shea S, Hripcsak G (2010). Accelerating the use of electronic health records in physician practices.. N Engl J Med.

[12] Wang X, Hripcsak G, Friedman C (2009). Characterizing environmental and phenotypic associations using information theory and electronic health records.. BMC Bioinformatics.

[13] Lobach DF, Detmer DE (2007). Research challenges for electronic health records.. Am J Prev Med.

[14] Gulliford MC, Charlton J, Ashworth M, Rudd AG, Toschke AM (2009). Selection of medical diagnostic codes for analysis of electronic patient records. Application to stroke in a primary care database.. PLoS One.

[15] Hivert MF, Grant RW, Shrader P, Meigs JB (2009). Identifying primary care patients at risk for future diabetes and cardiovascular disease using electronic health records.. BMC Health Serv Res.

[16] Li L, Chase HS, Patel CO, Friedman C, Weng C (2008). Comparing ICD9-encoded diagnoses and NLP-processed discharge summaries for clinical trials pre-screening: a case study..

[17] McCarthy EP, Iezzoni LI, Davis RB, Palmer RH, Cahalane M (2000). Does clinical evidence support ICD-9-CM diagnosis coding of complications?. Med Care.

[18] Goldstein LB (1998). Accuracy of ICD-9-CM coding for the identification of patients with acute ischemic stroke: effect of modifier codes.. Stroke.

[19] Keren R, Wheeler A, Coffin SE, Zaoutis T, Hodinka R (2006). ICD-9 codes for identifying influenza hospitalizations in children.. Emerg Infect Dis.

[20] Tsui FC, Wagner MM, Dato V, Chang CC (2001). Value of ICD-9 coded chief complaints for detection of epidemics..

[21] New York City Department of Health and Mental Hygiene (2010). NYC Influenza Information - Vaccination.. http://www.nyc.gov/html/doh/flu/html/vaccination/index.shtml.

[22] Glance LG, Osler TM, Mukamel DB, Meredith W, Wagner J (2009). TMPM-ICD9: a trauma mortality prediction model based on ICD-9-CM codes.. Ann Surg.

[23] Burd RS, Ouyang M, Madigan D (2008). Bayesian logistic injury severity score: a method for predicting mortality using international classification of disease-9 codes.. Acad Emerg Med.

[24] Zarychanski R, Stuart TL, Kumar A, Doucette S, Elliott L (2010). Correlates of severe disease in patients with 2009 pandemic influenza (H1N1) virus infection.. CMAJ.

[25] Centers for Disease Control and Prevention (2010). Patients hospitalized with 2009 pandemic influenza A (H1N1) - New York City, May 2009.. MMWR Morb Mortal Wkly Rep.

[26] Lombardo J, Burkom H, Elbert E, Magruder S, Lewis SH (2003). A systems overview of the Electronic Surveillance System for the Early Notification of Community-Based Epidemics (ESSENCE II).. J Urban Health.

[27] Centers for Disease Control and Prevention (2010). CDC Flu homepage.. http://www.cdc.gov/flu.

[28] Flahault A, Dias-Ferrao V, Chaberty P, Esteves K, Valleron AJ (1998). FluNet as a tool for global monitoring of influenza on the Web.. JAMA.

[29] World Health Organization (2010). WHO - FluNet.. http://www.who.int/globalatlas/autologin/flunet_login.asp.

[30] European Centre for Disease Prevention and Control (2010). European Influenza Surveillance Network (EISN).. http://ecdc.europa.eu/en/activities/surveillance/EISN/Pages/home.aspx.

[31] Kelly H, Grant K, Williams S, Smith D (2009). H1N1 swine origin influenza infection in the United States and Europe in 2009 may be similar to H1N1 seasonal influenza infection in two Australian states in 2007 and 2008.. Influenza Other Respi Viruses.

[32] Centers for Disease Control and Prevention (2009). H1N1 Early Outbreak and Disease Characteristics.. http://www.cdc.gov/H1N1FLU/surveillanceqa.htm.

[33] Khiabanian H, Farrell GM, St George K, Rabadan R (2009). Differences in patient age distribution between influenza A subtypes.. PLoS One.

[34] Olson DR, Heffernan RT, Paladini M, Konty K, Weiss D (2007). Monitoring the impact of influenza by age: emergency department fever and respiratory complaint surveillance in New York City.. PLoS Med.

[35] Ndifon W, Dushoff J, Levin SA (2009). On the use of hemagglutination-inhibition for influenza surveillance: surveillance data are predictive of influenza vaccine effectiveness.. Vaccine.

[36] Centers for Disease Control and Prevention (2004). Preliminary assessment of the effectiveness of the 2003-04 inactivated influenza vaccine--Colorado, December 2003.. MMWR Morb Mortal Wkly Rep.

[37] Ritzwoller DP, Bridges CB, Shetterly S, Yamasaki K, Kolczak M (2005). Effectiveness of the 2003-2004 influenza vaccine among children 6 months to 8 years of age, with 1 vs 2 doses.. Pediatrics.

