# Supplementary material for: Signs of the 2009 Influenza Pandemic in the New York-Presbyterian Hospital Electronic Health Records
Source: PLoS One. 2010 Sep 9;5(9):e12658. doi: 10.1371/journal.pone.0012658 (PMC2936568; doi:10.1371/journal.pone.0012658)
Supplement: Table S1 — ICD-9 codes associated with pandemic influenza, compared to seasonal (additional time windows). (0.09 MB DOC) [file pone.0012658.s001.doc]

**Supplementary Tables**

**Table S1: ICD-9 codes associated with pandemic influenza, compared to seasonal (additional time windows).**

| **Inquiry Interval (time pre/post flu)** | **ICD9 Codes *** | **Diagnoses** | **P-values †** | **FDR ‡** | **Ratio (Pandemic/Seasonal)** |
| --- | --- | --- | --- | --- | --- |
| 11 months (9/2) | **493.9** | **Asthma, unspecified** | **<0.001** | **<0.001** | **1.96** |
|  | **V12.61** | **Personal history of pneumonia** | **<0.001** | **0.015** | **6.31** |
|  | **348.39** | **Other encephalopathy** | **0.001** | **0.037** | **9.01** |
|  | 110.9 | Dermatophytosis… | 0.001 | 0.057 | 6.61 |
|  | 787.2 | Dysphagia, unspecified | 0.002 | 0.059 | 4.69 |
|  | 315.8 | Other specified delays in development | 0.004 | 0.130 | 3.20 |
|  | 709.8 | Other specified disorders of skin | 0.004 | 0.148 | 7.21 |
|  | 345.9 | Epilepsy unspecified | 0.005 | 0.147 | 2.52 |
|  | 789.59 | Other ascites | 0.006 | 0.210 | 10.8 |
|  | 577 | Acute pancreatitis | 0.007 | 0.236 | 3.97 |
|  | 262 | Other protein-calorie malnutrition | 0.010 | 0.275 | 6.31 |
|  | 338.29 | Other chronic pain | 0.010 | 0.275 | 6.31 |
|  | 599.7 | Hematuria, unspecified | 0.014 | 0.344 | 4.06 |
| 4 months (2/2) | **493.9** | **Asthma, unspecified** | **<0.001** | **<0.001** | **2.84** |
|  | **787.2** | **Dysphagia, unspecified** | **<0.001** | **0.011** | **16.2** |
|  | **V12.61** | **Personal history of pneumonia** | **<0.001** | **0.011** | **7.21** |
|  | **315.8** | **Other specified delays in development** | **0.001** | **0.025** | **5.41** |
|  | 262 | Other protein-calorie malnutrition | 0.002 | 0.053 | 12.6 |
|  | 493.92 | Asthma with acute exacerbation | 0.002 | 0.056 | 1.77 |
|  | 296.34 | Major depressive… disorder | 0.006 | 0.113 | 10.8 |
|  | 416.8 | Other chronic pulmonary heart diseases | 0.007 | 0.120 | 3.00 |
|  | 282.5 | Sickle-cell trait | 0.010 | 0.135 | 6.31 |
|  | 343.9 | Infantile cerebral palsy… | 0.010 | 0.135 | 6.31 |
|  | 348.39 | Other encephalopathy | 0.010 | 0.135 | 6.31 |
|  | 599.7 | Hematuria, unspecified | 0.012 | 0.151 | 4.81 |
|  | 493.91 | Asthma with status asthmaticus | 0.012 | 0.148 | 2.80 |
| same month | **493.9** | **Asthma, unspecified** | **<0.001** | **<0.001** | **2.61** |
|  | **315.8** | **Other specified delays in development** | **0.002** | **0.039** | **6.01** |
|  | **493.92** | **Asthma with acute exacerbation** | **0.003** | **0.041** | **1.90** |
|  | V12.61 | Personal history of pneumonia | 0.005 | 0.054 | 5.41 |
|  | 787.2 | Dysphagia, unspecified | 0.006 | 0.076 | 10.8 |
|  | 493.91 | Asthma with status asthmaticus | 0.007 | 0.082 | 3.60 |

* ICD codes and diagnoses lists include all p-values <0.015, excluding symptoms of influenza infection, and procedure-related supplemental (V) or external injury (E) codes (see Table 1 for other time windows and Supplemental Material Table S2 for all excluded ICD codes and diagnoses) † one-tail hypergeometric p-values, uncorrected; ‡ false discovery rate (FDR) described in Methods -- significant at FDR <0.05 (bolded)
