# Supplementary material for: Signs of the 2009 Influenza Pandemic in the New York-Presbyterian Hospital Electronic Health Records
Source: PLoS One. 2010 Sep 9;5(9):e12658. doi: 10.1371/journal.pone.0012658 (PMC2936568; doi:10.1371/journal.pone.0012658)
Supplement: Table S2 — Excluded ICD-9 codes associated with pandemic influenza, compared to seasonal. (0.13 MB DOC) [file pone.0012658.s002.doc]

**Supplementary Tables**

**Table S2: Excluded ICD-9 codes associated with pandemic influenza, compared to seasonal.**

| **Inquiry Interval (time pre/post flu)** | **ICD9 Codes *** | **Diagnoses** | **P-values †** | **FDR ‡** | **Ratio (Pandemic/Seasonal)** |
| --- | --- | --- | --- | --- | --- |
| 11 months (9/2) | 780.6 | Fever, unspecified | <0.001 | <0.001 | 9.59 |
|  | 786.2 | Cough | <0.001 | <0.001 | 2.48 |
|  | 487.1 | Influenza with other respiratory… | <0.001 | <0.001 | 1.06 |
|  | 780.96 | Generalized pain | <0.001 | 0.001 | 6.01 |
|  | V14.8 | Personal history of allergy… | <0.001 | 0.016 | 9.92 |
|  | 478.19 | Other disease of nasal cavity… | 0.001 | 0.051 | 2.43 |
|  | V04.81 | Need for… vaccination… influenza | 0.001 | 0.053 | 1.84 |
|  | V06.5 | Need for… vaccination… tetanus… | 0.002 | 0.101 | 12.6 |
|  | V05.3 | Need for… vaccination… hepatitis | 0.002 | 0.096 | 2.25 |
|  | 780.61 | Fever presenting with conditions… | 0.007 | 0.229 | 3.16 |
| 8 months (6/2) | 780.6 | Fever, unspecified | <0.001 | <0.001 | 10.6 |
|  | 786.2 | Cough | <0.001 | <0.001 | 2.69 |
|  | 487.1 | Influenza with other respiratory… | <0.001 | <0.001 | 1.07 |
|  | 780.96 | Generalized pain | <0.001 | 0.001 | 6.85 |
|  | V14.8 | Personal history of allergy… | <0.001 | 0.014 | 9.92 |
|  | 478.19 | Other disease of nasal cavity… | 0.001 | 0.027 | 2.58 |
|  | 79.99 | Unspecified viral infection | 0.001 | 0.055 | 1.34 |
|  | V06.5 | Need for… vaccination… tetanus… | 0.002 | 0.096 | 12.60 |
|  | V05.3 | Need for… vaccination… hepatitis | 0.003 | 0.114 | 2.37 |
|  | V04.81 | Need for… vaccination… influenza | 0.005 | 0.127 | 1.85 |
|  | 786.05 | Shortness of breath | 0.006 | 0.165 | 1.70 |
|  | 462 | Acute pharyngitis | 0.009 | 0.204 | 1.48 |
| 6 months (5/1) | 487.1 | Influenza with other respiratory… | <0.001 | <0.001 | 1.07 |
|  | 780.6 | Fever, unspecified | <0.001 | <0.001 | 10.5 |
|  | 786.2 | Cough | <0.001 | <0.001 | 2.86 |
|  | 780.96 | Generalized pain | <0.001 | 0.001 | 6.85 |
|  | V14.8 | Personal history of allergy… | <0.001 | 0.002 | 19.8 |
|  | 478.19 | Other disease of nasal cavity… | <0.001 | 0.009 | 2.97 |
|  | 79.99 | Unspecified viral infection | 0.003 | 0.112 | 1.32 |
|  | V14.0 | Personal history of allergy… | 0.004 | 0.109 | 3.91 |
|  | 784.1 | Throat pain | 0.005 | 0.133 | 5.41 |
|  | V06.5 | Need for… vaccination… tetanus… | 0.006 | 0.151 | 10.8 |
|  | V26.33 | Genetic counseling | 0.006 | 0.151 | 10.8 |
| 4 months (2/2) | 487.1 | Influenza with other respiratory… | <0.001 | <0.001 | 1.07 |
|  | 780.6 | Fever, unspecified | <0.001 | <0.001 | 10.7 |
|  | 786.2 | Cough | <0.001 | <0.001 | 3.50 |
|  | 780.96 | Generalized pain | <0.001 | <0.001 | 8.56 |
|  | 79.99 | Unspecified viral infection | <0.001 | <0.001 | 1.58 |
|  | 462 | Acute pharyngitis | <0.001 | 0.010 | 2.04 |
|  | 786.05 | Shortness of breath | <0.001 | 0.013 | 2.18 |
|  | V14.8 | Personal history of allergy… | 0.001 | 0.019 | 14.4 |
|  | 784 | Headache | 0.001 | 0.018 | 1.77 |
|  | V72.6 | Laboratory examination | 0.003 | 0.053 | 2.29 |
|  | 478.19 | Other diseases of nasal cavity… | 0.005 | 0.081 | 2.58 |
|  | 787.03 | Vomiting alone | 0.005 | 0.077 | 1.76 |
|  | V14.0 | Personal history of allergy… | 0.006 | 0.118 | 3.97 |
|  | 780.61 | Fever presenting with conditions… | 0.007 | 0.118 | 3.60 |
| same month | 487.1 | Influenza with other respiratory… | <0.001 | <0.001 | 1.08 |
|  | 780.6 | Fever, unspecified | <0.001 | <0.001 | 11.0 |
|  | 786.2 | Cough | <0.001 | <0.001 | 4.50 |
|  | 780.96 | Generalized pain | <0.001 | <0.001 | 8.56 |
|  | 784 | Headache | <0.001 | 0.007 | 1.95 |
|  | V14.8 | Personal history of allergy… | <0.001 | 0.013 | 14.4 |
|  | 462 | Acute pharyngitis | 0.002 | 0.029 | 2.01 |
|  | 79.99 | Unspecified viral infection | 0.003 | 0.037 | 1.42 |
|  | V72.6 | Laboratory examination | 0.004 | 0.043 | 3.38 |
|  | 787.03 | Vomiting alone | 0.004 | 0.040 | 2.06 |
|  | 786.05 | Shortness of breath | 0.007 | 0.079 | 2.00 |

* Excluded from Table 1 and Table S1 were all ICD codes and diagnoses related to symptoms of influenza infection, procedure-related supplemental (V) or external injury (E) codes † one-tail hypergeometric p-values, uncorrected; ‡ false discovery rate (FDR) described in Methods -- significant at FDR <0.05
